# Sugarcane stem node detection and localization for cutting using deep learning

**DOI:** 10.3389/fpls.2022.1089961

**Published:** 2022-12-12

**Authors:** Weiwei Wang, Cheng Li, Kui Wang, Lingling Tang, Pedro Final Ndiluau, Yuhe Cao

**Affiliations:** ^1^ School of Engineering, Anhui Agricultural University, Hefei, China; ^2^ Anhui Intelligent Agricultural Machinery and Equipment Engineering Laboratory, College of Engineering, Anhui Agricultural University, Hefei, China

**Keywords:** sugarcane seed cutting, enhanced YOLOv4-Tiny, sugarcane stem node, identification system, cutting system

## Abstract

**Introduction:**

In order to promote sugarcane pre-cut seed good seed and good method planting technology, we combine the development of sugarcane pre-cut seed intelligent 0p99oposeed cutting machine to realize the accurate and fast identification and cutting of sugarcane stem nodes.

**Methods:**

In this paper, we proposed an algorithm to improve YOLOv4-Tiny for sugarcane stem node recognition. Based on the original YOLOv4-Tiny network, the three maximum pooling layers of the original YOLOv4-tiny network were replaced with SPP (Spatial Pyramid Pooling) modules, which fuse the local and global features of the images and enhance the accurate localization ability of the network. And a 1×1 convolution module was added to each feature layer to reduce the parameters of the network and improve the prediction speed of the network.

**Results:**

On the sugarcane dataset, compared with the Faster-RCNN algorithm and YOLOv4 algorithm, the improved algorithm yielded an mean accuracy precision (MAP) of 99.11%, a detection accuracy of 97.07%, and a transmission frame per second (fps) of 30, which can quickly and accurately detect and identify sugarcane stem nodes.

**Discussion:**

In this paper, the improved algorithm is deployed in the sugarcane stem node fast identification and dynamic cutting system to achieve accurate and fast sugarcane stem node identification and cutting in real time. It improves the seed cutting quality and cutting efficiency and reduces the labor intensity.

## 1 Introduction

**Figure 1:**
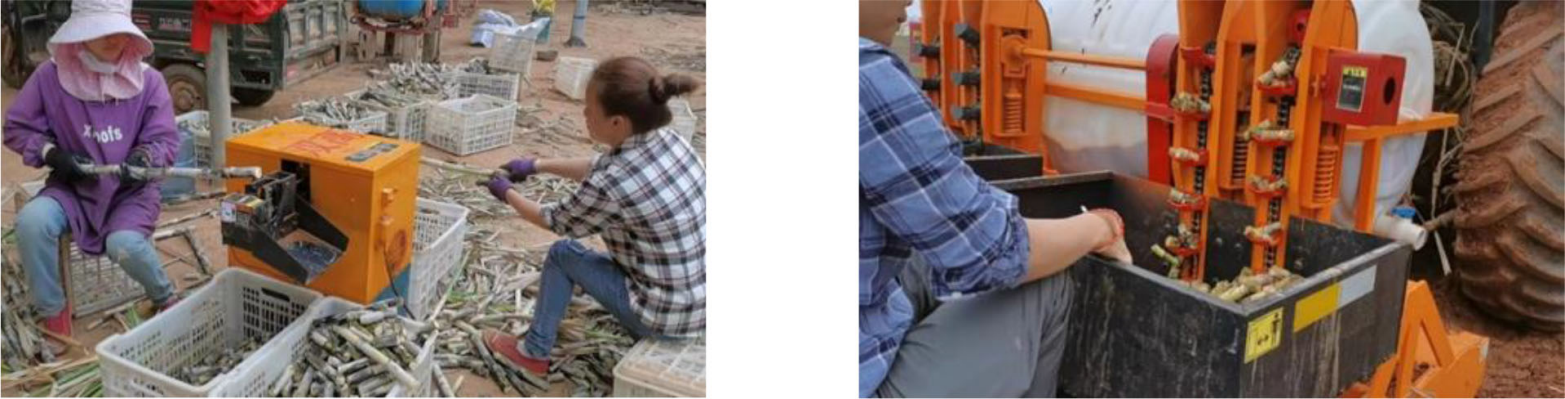
Domestic sugarcane cutting method.

Sugarcane is one of the important sources of sugar and production fuel (Zhenfeng et al, 2022), which is crucial to secure people’s livelihood. Most of the existing sugarcane seeders are real-time cane feeding type seeding, which requires pre-cutting of sugarcane stems and nodes. The existing manual seed cutting method not only has low seed cutting quality and high cane bud loss, but also has high labor intensity (**Figure 1**) and low production efficiency. Therefore, the development of intelligent sugarcane pre-cutting machinery and equipment is of great significance to improve the efficiency of sugarcane production.

The key to achieve automatic seed cutting of sugarcane is to identify the location of the stem nodes of sugarcane. In recent years, many scholars have explored the use of digital image processing techniques to identify the stem nodes of sugarcane. For example (Yiqi et al., 2017), in order to realize the automatic cutting of single bud segment of sugarcane, the average grayscale of sugarcane image is calculated on HSV color space for the mean filtered processed sugarcane image, and the position of the maximum grayscale value is considered as the stem node position. Zhang et al. extracted stem node features based on hyperspectral imaging and identified stem nodes, obtaining a high accuracy rate (Weizheng et al., 2017). However, this method requires expensive hyperspectral data acquisition equipment and is not suitable for field use. Shi et al. proposed a machine vision-based stem node recognition method to solve the problem of sugarcane species diversity (Changyou et al., 2019). This algorithm can accurately identify the stem nodes of diverse types of sugarcane under different background conditions. However, too complex sugarcane images can also reduce the stem node recognition rate. Yang Rui et al. proposed a method for simultaneous recognition of multiple stem nodes of sugarcane based on the characteristic of obvious color change of leaf marks above and below the stem nodes, which improved the detection efficiency. However, when the degree of color variation of wax powder at a certain place is similar to that of the stem epidermis, the wax powder will be mistaken for sugarcane nodes (Rui et al., 2020). In order to achieve damage-proof budding and automatic cutting of single bud segments of sugarcane, Yang Changhui et al. used leaf marker features to identify sugarcane nodes and constructed a feature vector describing the sugarcane image (Changhui et al. (2019). The locations of all stem nodes in the sugarcane image were searched by defining the values of vector elements at the sugarcane nodes and the distance between the sugarcane nodes. This machine vision-based sugarcane cutting system can achieve a recognition rate of 93% and the average recognition time is only 0.539 s. However, in reality, many black or white powders are attached to the surface of sugarcane, which can cause great interference to the processed images. Identification of sugarcane nodes using images may also suffer from limited speed, low recognition efficiency and high cost, making it difficult to be applied in a practical production environment.

With the rapid development of deep learning, many neural network algorithms are applied to the recognition of agricultural products with good results (Aichen et al., 2019; Shangping et al., 2019; Shiping et al., 2020; Xu et al., 2021; Arunabha M and Bhaduri, 2022; Masum et al., 2022). Neural network recognition algorithms use two-stage and one-stage strategies. To improve recognition accuracy, two-stage neural networks are usually used to recognize agricultural products (Aichen et al., 2020; Chen et al., 2020; Fangfang et al., 2020; Subramanian and Selvi, 2021; Shenglian et al., 2022). Jia et al. proposed a mask region convolutional neural network (Mask R-CNN) based picking robot vision detector model with an accuracy of 97.31% and a recall rate of 95.70% (Weikuan et al., 2020). To improve the speed of target recognition, researchers often use single-stage neural networks to identify agricultural products (Dihua et al., 2020; Xiaoyu et al., 2021; Xuelong et al., 2021). Shi et al. proposed a generalized attribute method for pruned detection networks that can be easily fine-tuned to accurately detect mangoes in real time (Rui et al., 2020), reducing the computational effort of pruning detection networks by 68.7% while improving accuracy by 0.4% compared to unpruned fine-tuned networks. With the refinement of deep learning methods, researchers began to apply them to sugarcane detection. Song et al. proposed a convolutional neural network for sugarcane bud classification that can classify buds into good and bad buds (Huaning et al., 2021). Chen et al. proposed a deep learning-based target detection algorithm to achieve accurate identification of sugarcane stem nodes under the data expansion and lighting condition in different time periods (Wen et al., 2021). These algorithms mentioned above are based on the conditions that the sugarcane stem nodes are normal and the light intensity is good at the time of recognition. The accuracy of the identification of sugarcane stem nodes will be affected when they are broken or covered with soil, as well as when the light is dim. Also, due to the limitation of equipment, these methods are difficult to be used for real-time detection in the field.

These methods mentioned above have improved the recognition accuracy of sugarcane stem node, but the following problems are still unsolved: (1) After the sugarcane is harvested, some of the stems and node are broken or covered with soil. How to recognize such stem node is still a difficult problem; (2) At present, in order to achieve high recognition rate of sugarcane stem node in the natural environment, the model occupies large memory and requires high computing power and memory of the device, which is not applicable to embedded devices; (3) The recognition efficiency is low and time-consuming. In order to achieve accurate and fast real-time recognition and cutting of sugarcane stem node, this paper designs a real-time sugarcane stem node detection and recognition system based on the enhanced YOLOv4-Tiny network model according to the sugarcane stem node feature information. The enhanced YOLOv4-Tiny network model structure is used to collect sugarcane stem node information. The detection and recognition system transmits the sugarcane information to the cutting system in real time to realize the fast cutting of sugarcane stem node.

## 2 Related work

### 2.1 Sugarcane object

**Figure 2:**
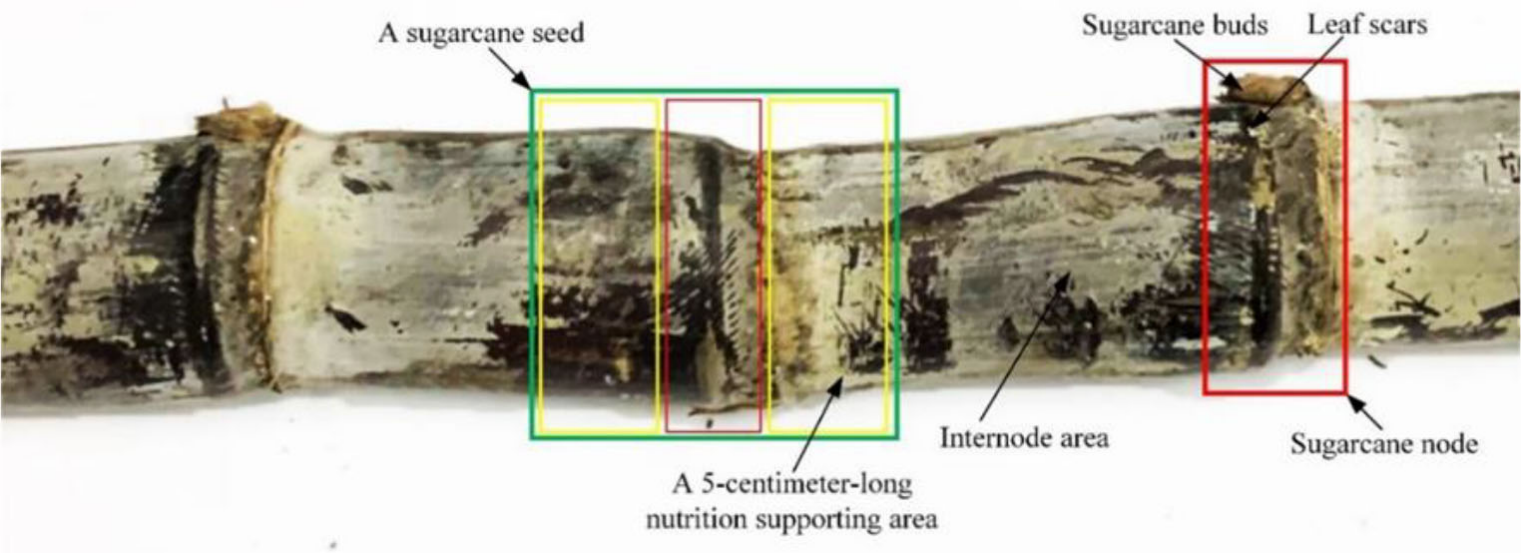
Description of sugarcane seed cutting requirements.

In this paper, sugarcane was studied by stripping the sugarcane stem leaves leaving the sugarcane stem, which consists of the internode area and the stem node area, as shown in **Figure 2**. Sugarcane buds and leaf scars were found in the sugarcane stem node, and only one bud was found in a sugarcane stem node. The sugarcane buds were located on the upper side of the stem leaf scars near the tip of the sugarcane, and the sugarcane stem buds were not necessarily present when the sugarcane stem node information was collected using the monocular camera. The leaf scars surround the cane stem for one week, and the leaf scars show up more clearly in the image. The equipment designed in this paper can be used to cut the sugarcane stem node into segments, with only one stem node per segment and a 5-cm-long internode on each side of the stem node to provide nutrients to the sugarcane seed later.

### 2.2 System overall scheme

**Figure 3:**
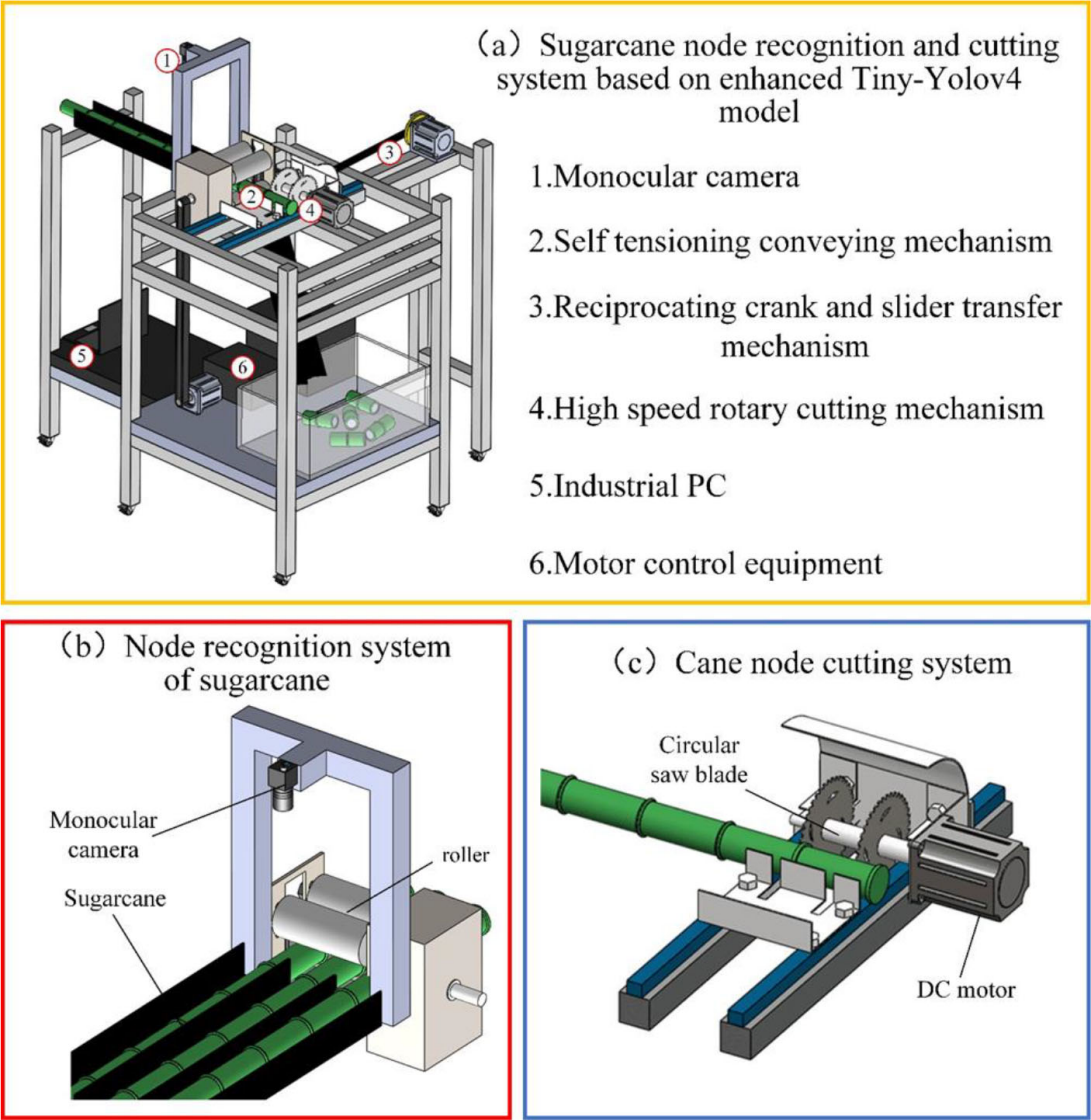
Design overview of seed cutting mechanism. **(A)** Sugarcane cutting device **(B)** Identification system **(C)** Cutting system.

To meet the seed cutting requirements, the design of the seed cutting device is outlined in **Figure 3**; Wang et al., 2022, which includes the cane stem node target detection system and the cane stem node cutting system; the cane stem node target detection system is mainly composed of monocular camera, Industrial Personal Computer(IPC), switching power supply, etc.; the cane stem node cutting system is mainly composed of self-tensioning conveying mechanism, reciprocating crank slider transfer mechanism and high-speed rotary cutting mechanism, which complete the processes of conveying and cutting of cane respectively.

The basic principle of the system can be described as follows: when the sugarcane section cutting system works, the seed cane is put into the feeding guide and pushed inward until it is bitten by the upper and lower rollers, at which time the STM32 microcontroller controls the stepper motor1 to drive the rollers to transport the seed cane inward. When the sugarcane stems reach the lower end of the monocular camera, the camera acquires the sugarcane stems and extracts the information of sugarcane stems after processing and matches the extracted stems with the sample training library and then carries out segment detection and calibration to generate the target detection frame, and then passes the distance information of the adjacent target detection frame to the STM32 microcontroller through CAN communication. The STM32 microcontroller converts the distance information into a certain number of pulses of stepper motor 1, stops when stepper motor 1 rotates the corresponding number of pulses, and controls stepper motor 2 to rotate once to drive the cutting mechanism to reciprocate once to complete the cutting action. In this process, the DC brushless motor always drives the circular saw blade to maintain a high-speed rotation. The cut sugarcane stem node slides down the discharge guide into the collection frame.

### 2.3 Image recognition system

**Figure 4:**
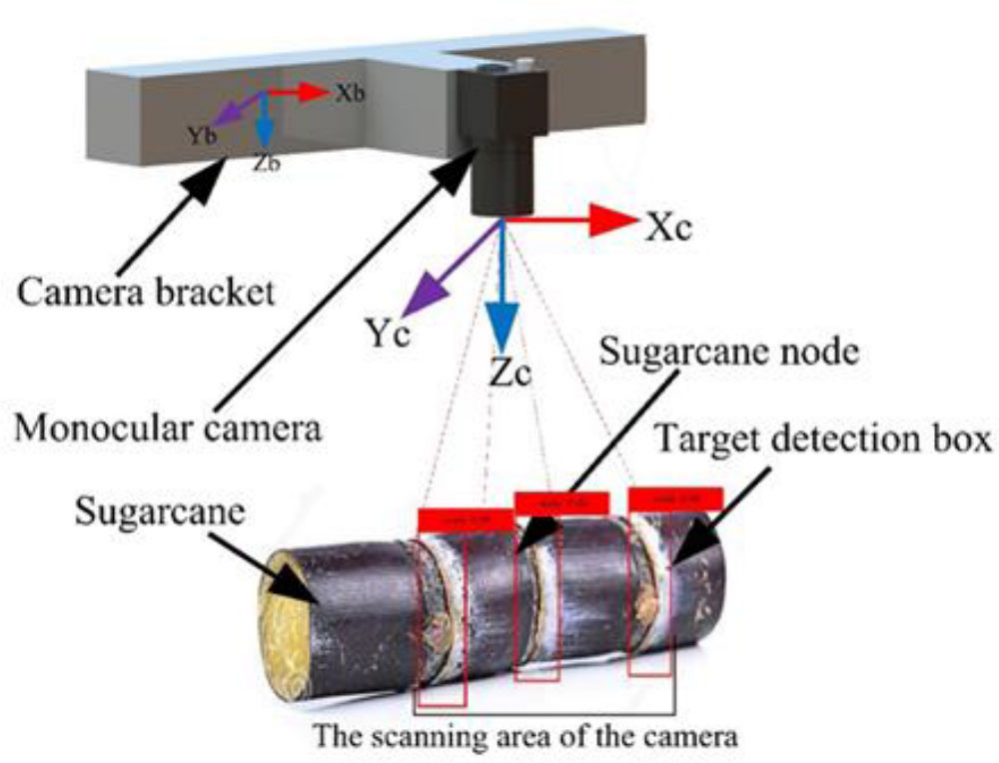
Schematic diagram of image acquisition.

On the sugarcane machine stand at Anhui Agricultural University’s Mechanical and Electrical Engineering Park, a monocular camera of type MV-SUA502C/M-T was used to acquire sugarcane stem node images of size 1280 × 960 with white as the background. The monocular camera has a lens focal length of 8mm, a maximum resolution of 2592×1944, and a lens-to-cane height distance of 500mm (**Figure 4**). PyCharm2020.3 was used for image processing, and black-skinned sugarcane was used as the test material. A total of 3000 images were collected from sugarcane under different conditions such as different light, with soil and stem node damage. The images captured by the monocular camera are uploaded to the target detection system, and the cutting information is transmitted to the cutting system after a series of operations such as calibration, identification, and detection. The model used in this study is based on an enhanced version of YOLOv4-Tiny, which can accurately and quickly identify and locate sugarcane stem nodes, ensuring the quality and efficiency of seed cutting.

### 2.4 Dataset production

**Figure 5:**
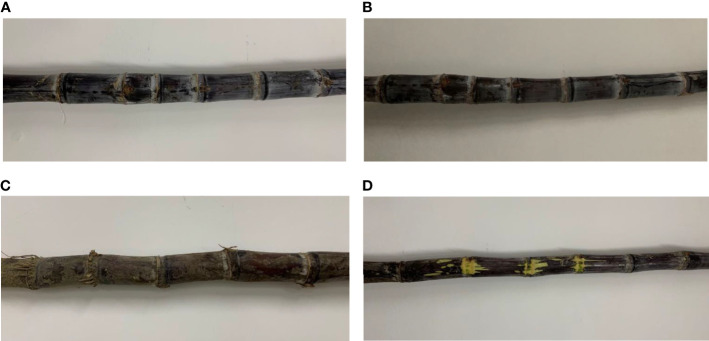
Sugarcane samples. **(A)** Images captured with normal lighting **(B)** Images taken under dark light **(C)** Image of sugarcane with dirt **(D)** Image of stem node damage.

In order to enrich the image dataset, better extract the sugarcane stem node features and improve the generalization ability of the model, OpenCV was used to augment the data of the original sugarcane dataset. The rotation angle is randomly taken as 45° and 135°, and the original image is randomly mirrored flipped, horizontally flipped and vertically flipped, cropped and scaled to extend the dataset. The data are enhanced by image processing techniques such as adjusting saturation and hue, histogram equalization, and median filtering. The final dataset has a total of 15,000 images. (**Figure 5**).

To ensure the correspondence between labels and data and the uniform distribution of the dataset, the enhanced dataset is randomly divided into a training set and a test set in the ratio of 9:1. The final data set was stored in the format of PASCAL VOC dataset, with 13499 samples in the training set and 1501 samples in the test set. The training set samples include 10,147 normal sugarcane stem node images, 1,552 broken sugarcane stem node images and 1,800 muddy sugarcane stem node images. The final dataset is shown in **Table 1**.

**Table 1:** Sugarcane sample quantity.

Dataset	Normal sugarcane	Damaged sugarcane	Sugarcane with mud
Training set	10147	1552	1800
Test set	1128	173	200
Total number	11275	1725	2000

### 2.5 Model improvements

#### 2.5.1 Enhanced YOLOv4-Tiny model structure

In agriculture, since the target detection system is limited by the mobile platform, the size of the algorithm is usually limited and the detection speed is restricted in order to meet the real-time detection demand. To solve this problem, we found that YOLOv4-Tiny has only one-tenth of the training parameters of YOLOv4 and the model is loaded faster, while the measured speed is about 22fps, which is suitable for field detection. Of course fewer parameters and faster speed are traded for accuracy. In order to improve the accuracy, we improved the YOLOv4-Tiny model.

**Figure 6:**
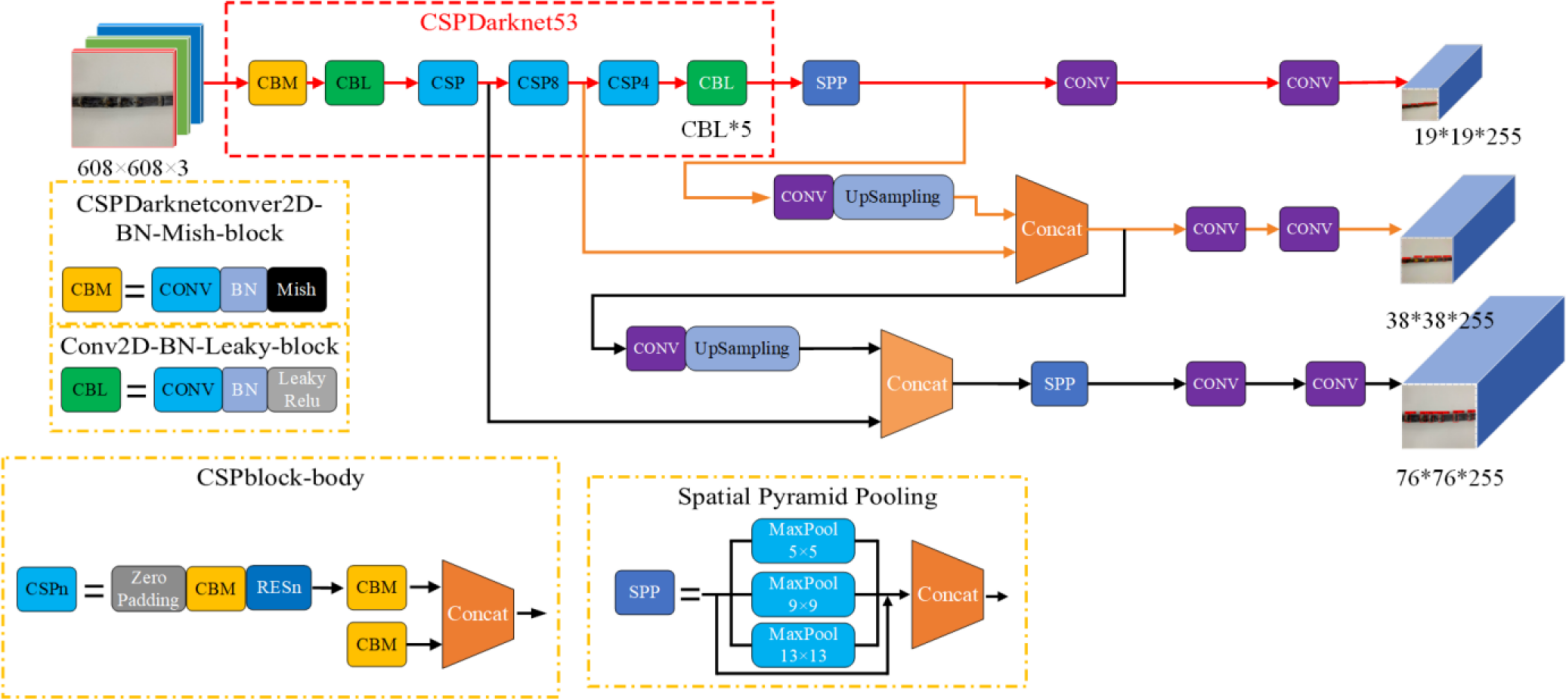
Enhanced YOLOv4-Tiny network structure.

The new model uses the same data enhancement method as YOLOv4 on the input side, which increases the training data, improves the generalization ability of the model, and avoids model overfitting (Fu et al., 2021). The enhanced YOLOv4-Tiny uses the CSPDarknet53 (**Figure 6**) as the backbone feature extraction network. The feature extraction network consists of a CBM module, two CBL modules and three CSPn modules. The CBM module consists of convolution, batch normalization and the Mish activation function, where the Mish function has better prediction accuracy than the Leaky_ReLU function. The CBL module is the same as YOLOv4-Tiny, consisting of convolution, batch normalization and the Leaky_ ReLU function. The improved model uses the Mish activation function only in the first step of Backbone logic calculation, and the Leaky_ReLU activation function is still used later in the network, preserving the detection speed advantage of YOLOv4-Tiny and improving the detection accuracy. To obtain faster detection speed, the CSPn structure is added to the model. This structure is borrowed from the CSPNet structure and consists of an all-zero padding, three convolutional layers, and n Res unint modules Concat. It solves the problem of requiring a large number of inference calculations, reducing the computation by 20% and reducing the memory footprint while maintaining the same or even higher accuracy, allowing the model to be applied to embedded devices.

In the field of target detection, for better extraction of fusion features, usually in the Backbone and output layers, some layers are inserted, and this part is called Neck. It is equivalent to the neck of the target detection network and is also very critical. Different from the original FPN network of YOLOv4-Tiny, in the Neck part of this paper, the original PANet structure with the same number of channels is chosen to be used in order to optimize the memory access and usage, while the cat operation of the original network is reduced to an add operation. Also, a bottom-up spatial pyramid pooling (SPP) is added behind the FPN layer. SPP uses 1×1, 5×5, 9×9, and 13×13 maximum pooling for multi-scale fusion. It performs a direct fixed-size pooling of feature maps of arbitrary size to obtain a fixed number of features. Each pooled feature is then combined to obtain a fixed number of features of fixed length (the dimensionality of the feature map is fixed), which can then be fed into the fully connected layer for training the network. In this way, the FPN layer conveys strong semantic features (High-Level features) from the top down, while the feature pyramid conveys shallow features (Low-Level features) from the bottom up. The aggregation of parameters from different backbone layers to different detection layers further improves the feature extraction capability and improves the recognition of broken and mud-stained sugarcane stems and nodes.

#### 2.5.2 Evaluation index

In order to accurately assess the performance of the model, Precision, Recall and Average Precision are used as the evaluation metrics for sugarcane stem node identification. Recall refers to the probability that the predicted outcome is also a positive sample (including the predicted negative sample, but the actual positive sample) among all the actual positive sample outcomes; Average Precision refers to the area of the P-R curve using different combinations of Precision and Recall values. The larger the MAP value is, the better the model effect is. the formula of Precision and Recall is:

Recall=TPTP+FN (1)

$\mathrm{Pr}ecision=\frac{TP}{TP+FP}$ (2)

$MAP=\int_{0}^{1}(PR)dR$ (3)

Where TP is the number of positive samples detected, i.e., the number of stem node samples that were correctly detected; TN is the number of negative samples detected, i.e., the other parts of the cane that were not boxed; FN is the number of positive samples detected as negative samples, i.e., the number of stem nodes that were not detected; and FP is the number of negative samples detected as positive samples, i.e., the other areas of the cane that were detected as stem nodes. Recall and accuracy are based on a threshold of 0.5, and both AP and F1 scores can be used to evaluate the performance of the target detection model, with AP being the area under the PR curve.

## 3 Results and discussion

### 3.1 Model training

**Figure 7:**
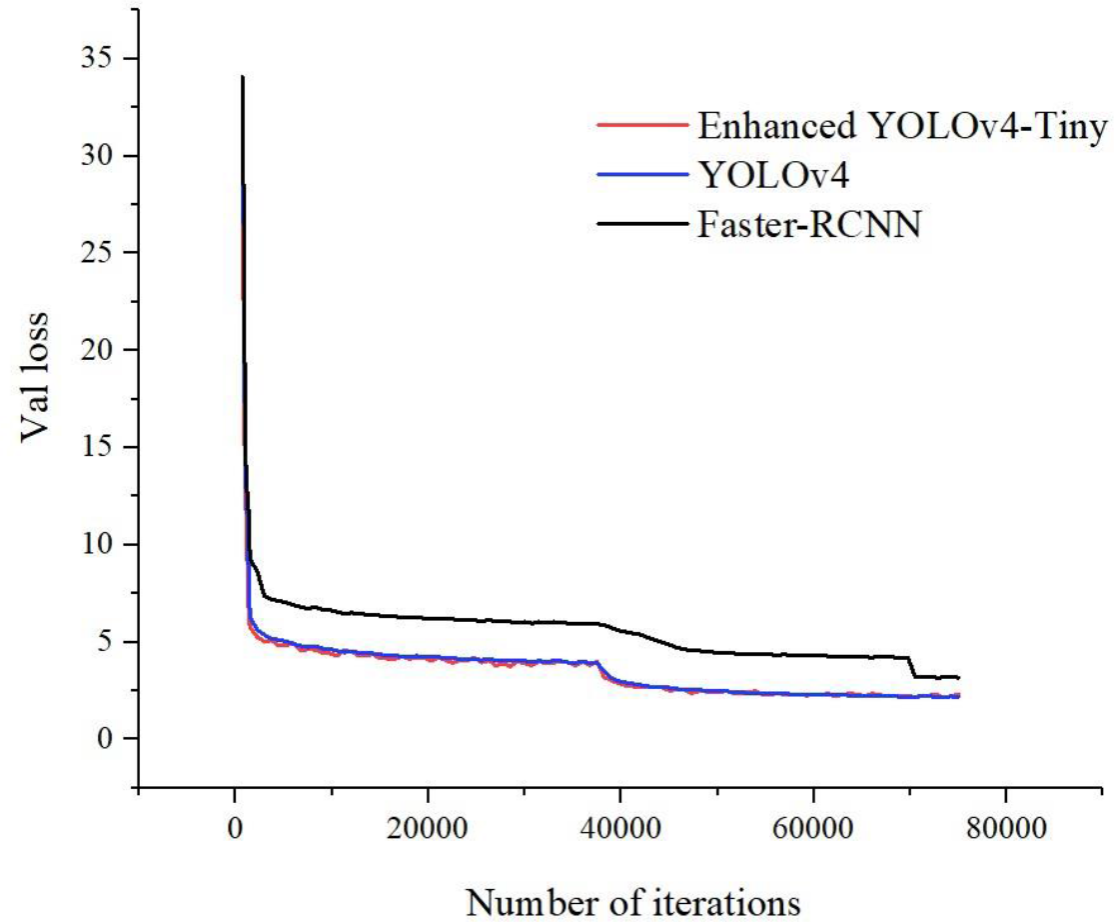
Model training loss value change trend diagram.

The loss value is one of the metrics to measure the effectiveness of model training. Theoretically, the smaller the loss value is, the better the training effect of the model is. **Figure 7** shows the loss curves during the training period, where different colors represent different models. From **Figure 7**, it can be seen that the model learns more efficiently and the training curve converges faster in the initial stage of the training of the sugarcane stem node detection model. After 750 iterations, the model loss value of the enhanced YOLOv4-Tiny rapidly converges to below 5.5 and becomes stable after 40,500 iterations. With further training, the slope of the training curve gradually decreases. Throughout the training process, Faster-RCNN tested higher loss values than other models. The enhanced YOLOv4-Tiny loss curve is very close to YOLOv4, but the fluctuations are smaller than YOLOv4. enhanced YOLOv4-Tiny converges in a shorter time compared to YOLOv4. This is because YOLOv4 has more convolutional layers of the network and requires more time to learn. The enhanced YOLOv4-Tiny network model decreases the training set loss (total loss) and test loss (Val loss) as the number of iterations increases. Finally, when the number of training iterations reaches about 40500, the learning efficiency of the enhanced YOLOv4-Tiny model gradually reaches saturation, and the total loss and test loss values gradually converge, and the final loss value is stabilized at about 2, which proves that the training results are good.

### 3.2 Model comparative analysis

**Figure 8:**
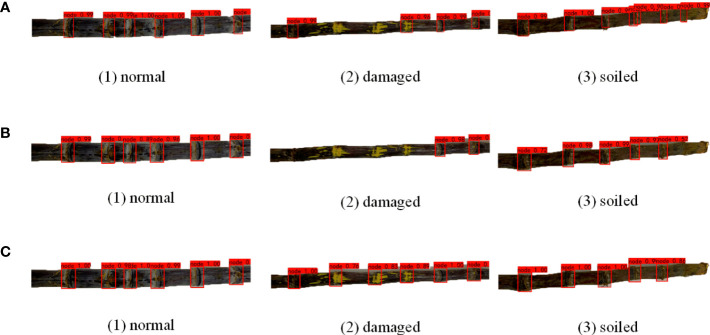
Sugarcane target recognition under different models. **(A)** Faster RCNN **(B)** YOLOv4 **(C)** Enhanced YOLOv4-Tiny.

To verify the effectiveness and advantage of the enhanced YOLOv4-Tiny sugarcane detection network proposed in this study for sugarcane target stem node recognition in complex situations, the current representative target detection networks Faster-RCNN and YOLO4 were trained with the same dataset and training parameters for the model and tested on the test set for comparison. Normal sugarcane, broken sugarcane and sugarcane with soil are selected in the test set for comparative recognition detection, and the results of the detection are compared as shown in **Figure 8**.

From **Figure 8**, it can be seen that for sugarcane under normal conditions, Faster-RCNN, YOLOv4 and the enhanced YOLOv4-Tiny model proposed in this paper can identify all six sugarcane stem node targets. Only Faster-RCNN could identify all the six stem node targets of sugarcane with soil, and both YOLOv4 and enhanced YOLOv4-Tiny had missed detection and did not identify the stem node target closest to the root. In identifying the fifth stem node from the left, the confidence level of the YOLOv4 model is only 0.52 and that of the enhanced YOLOv4-Tiny model is only 0.55, both very close to the threshold. The model Faster-RCNN, on the other hand, showed repeated marking in identifying the fourth stem node from the left, incorrectly marking leaf scars and wax powder as sugarcane stem node. In identifying the six stem node targets with broken sugarcane node, the enhanced YOLOv4-Tiny target detection network can identify all six stem node targets, and the Faster-RCNN detection network can identify four of the six sugarcane stem node targets. YOLOv4 performs the worst, identifying only two of the six targets. Due to the small feature area of the broken stem node, less information can be reflected in the fixed area, and much feature information of the stem node has been lost after multiple down sampling during feature extraction by the convolutional neural network, resulting in the missed detection of stem targets. The enhanced YOLOv4-Tiny also adds a bottom-up feature pyramid behind the FPN layer, which contains two PAN structures. This allows parameter aggregation of different detection layers from different backbone layers to further improve the feature extraction capability. Experiments show that the enhanced YOLOv4-Tiny performs better for the detection of broken sugarcane stem node.

In the actual sugarcane cutting environment, sugarcane stem node is often damaged and sticky due to the environment and harvesting method, which makes detection and identification difficult. The enhanced YOLOv4-Tiny target detection network proposed in this paper can correctly identify the stem node targets in the case of breakage with high recognition rate and can also achieve 84.4% recognition rate for the stem node targets with soil. In contrast, the Faster-RCNN and YOLOv4 detection networks have a lower recognition rate for broken sugarcane targets. The training results show that the enhanced YOLOv4-Tiny is more accurate than the other two models in detecting stem-node targets in different situations.

**Figure 9:**
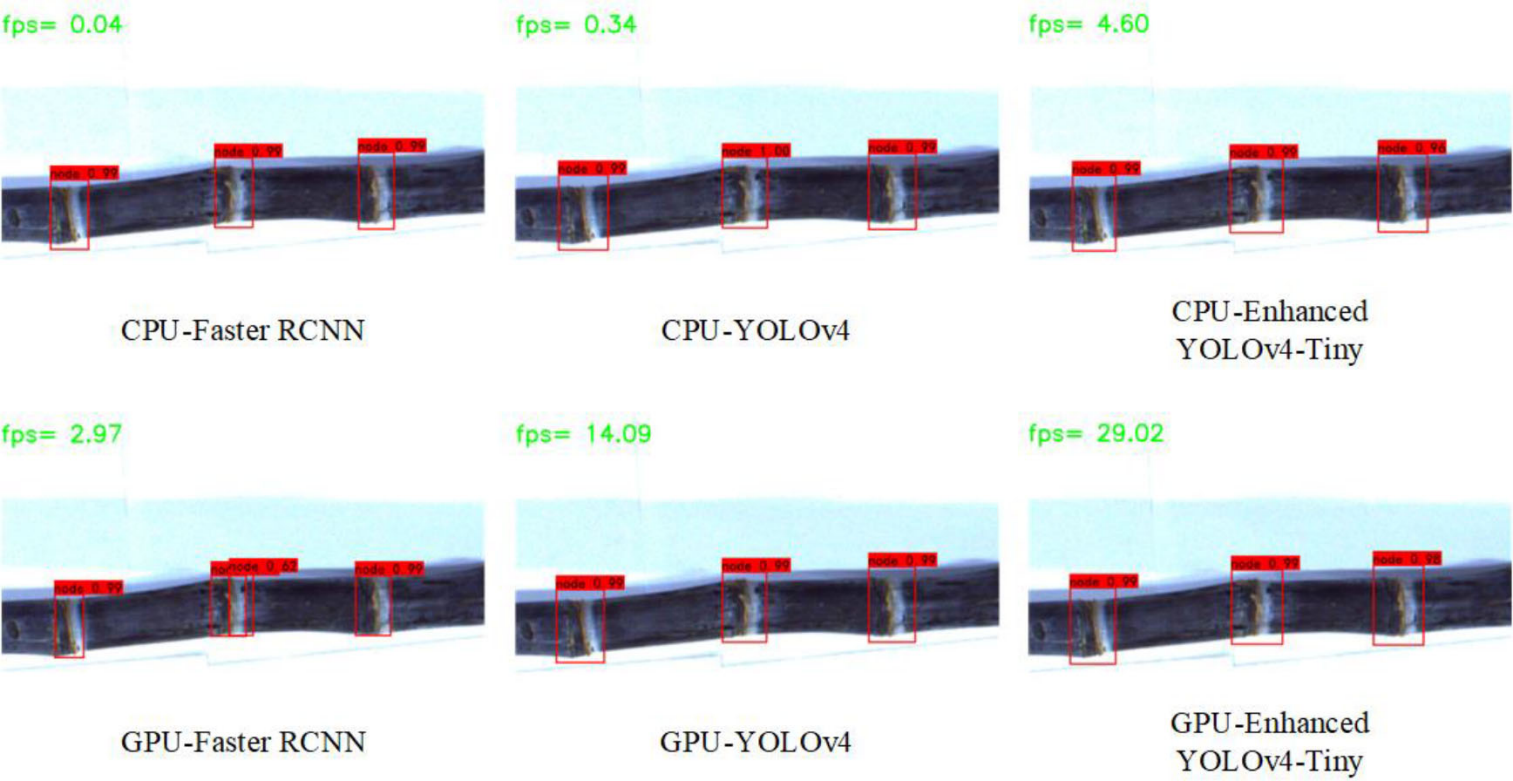
Recognition speed of sugarcane targets in different models under CPU and GPU.

As can be seen in **Figure 9**, the enhanced YOLOv4-Tiny can achieve a detection speed of 4.60 fps with the same CPU computation, which is 13.5 times faster than Yolov4 and 115 times faster than Faster-RCNN. With the same GPU computing, the enhanced YOLOv4-Tiny can achieve a detection speed of 29.02 fps, which is nearly 2 times faster than YOLOv4’s detection speed and nearly 10 times faster than Faster-RCNN. The enhanced YOLOv4-Tiny has significantly improved detection speed compared to other models under CPU computing and GPU computing, respectively, and also ensures detection accuracy. The detection speed of the model is much higher than that of the CPU under GPU computing, where the detection speed of Faster-RCNN under GPU computing is nearly 75 times faster than that of the CPU, and the detection speed of YOLOv4 under GPU computing is 41 times faster than that of the CPU. The speed of YOLOv4-Tiny is 29.02 frames/s under GPU computing and 4.60 frames/s under CPU computing, a difference of six times. Since the size of the model proposed in this paper is only 48MB, the complexity of the model is greatly reduced while ensuring the detection accuracy, and the advantage of GPU computing is not obvious. However, the GPU was initially designed to handle the rendering of graphic images, using a large number of small cores to operate simultaneously to speed up the operation. For the same amount of time, the training error can be reduced to an acceptable value with the GPU, while the training error remains high with the CPU.

In this paper, the test results of the validation set were statistically analyzed using the formula in Section 2.5.2, and the results are shown in **Table 2**. The accuracy, detection speed and MAP of the improved model are improved to different degrees. The detection accuracy of the enhanced YOLOv4-Tiny model reaches 97.07%, which is 35.89% higher than Faster-RCNN and 8.94% higher than YOLOv4. In terms of processing accuracy, the dataset in this study is manually captured images. Therefore, the background information is relatively simple. Under slightly more complex background conditions, the accuracy may be reduced. Similarly, the enhanced YOLOv4-Tiny model has a higher MAP value of 99.11% than the Faster-RCNN model value of 95.19% and the YOLOv4 model value of 90.73%. Along with the increase in accuracy and MAP, the enhanced YOLOv4-Tiny model also improves the average arithmetic speed. The average arithmetic speed of the enhanced YOLOv4-Tiny is 30 frames/s, which is twice that of YOLOv4 and 10 times that of Faster-RCNN. This significantly improves the detection speed of the model, which can recognize more stem node images and detect more sugarcane the same time, greatly improving the work efficiency. The enhanced YOLO4-Tiny model achieves 97.07% detection accuracy and 98.46% recall, which is a very small compared with 98.85% of Faster-RCNN and 98.62% recall of YOLOv4. Based on the guaranteed accuracy, the complexity of the model is an important factor affecting the detection speed. The complexity of the enhanced YOLOv4-Tiny model is greatly reduced, and the size of the improved model is only 48MB, which is much smaller than the 265MB of Faster-RCNN and 245MB of YOLOv4, and it is a lightweight detection network, which is suitable for embedded development, and the model inference speed also ensures the feasibility of real-time detection and is suitable for agricultural platform. The results of the experiments show that the enhanced YOLOv4-Tiny network proposed in this paper with high accuracy and speed.

**Table 2:** Comprehensive comparison of different detection networks for sugarcane stem node detection.

Network Model	Precision/%	Recall/%	Mean operation Rate/%	Model size/MB	MAP/%
Faster-RCNN	61.18	98.85	3	265	90.73
YOLOv4	88.13	98.62	15	245	95.19
Enhanced YOLOv4-Tiny	97.07	98.46	30	48	99.11

**Figure 10:**
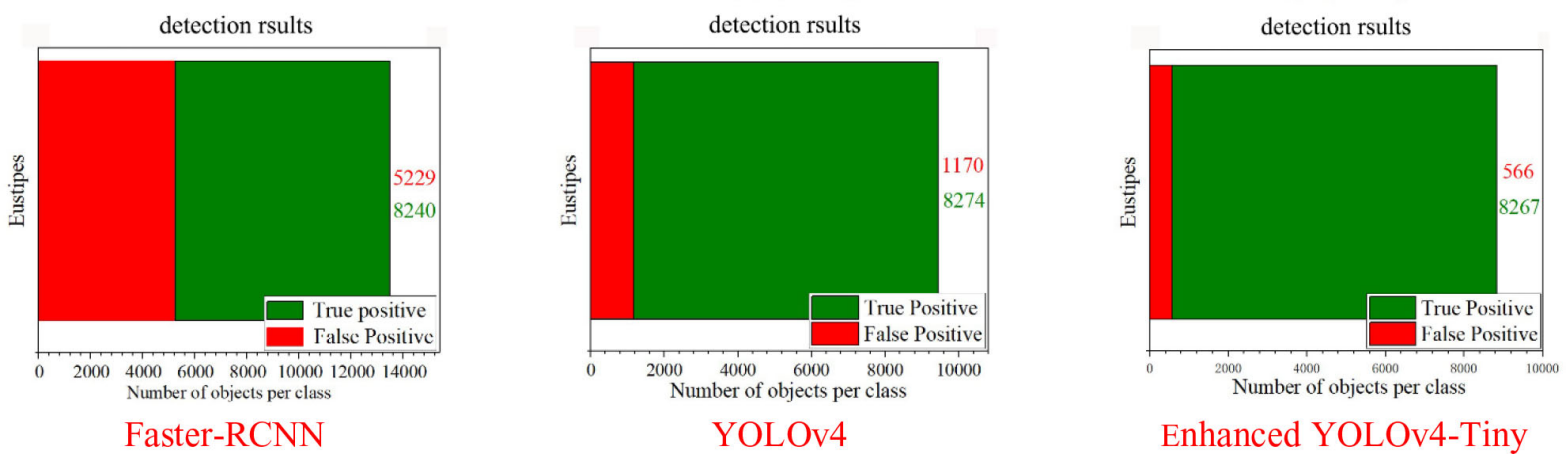
Identification and detection effect of sugarcane stem segment under different models.

To clarify the predicted results of the 3 models for the sugarcane categories under different scenarios, the values of the indicators in the above table are represented using bar charts:

Combined analysis of the bar comparison graphs of the above three model evaluation metrics, for **Figure 10**, the green region True Positives refers to samples that are correctly detected by the model and predicted to be positive classes with the intersection ratio greater than the threshold; the red region False Positives refers to false detections, which are predicted to be positive by the model, but the intersection ratio is less than the threshold or incorrectly classified. The larger the green area is relative to the red area, the better the model detection is. Among the three models, for the same number of targets, the Faster-RCNN target detection network correctly detected the sugarcane target 8240 times and incorrectly detected 5229 times, with a ratio of 1.57; the YOLOv4 detection network correctly detected 8274 times and incorrectly detected 1170 times, with a ratio of 7.07; while the enhanced YOLOv4-Tiny model proposed in this paper can correctly detect the cane 8267 times correctly and 566 times incorrectly, with a ratio as high as 14.61, which is much higher than the other two detection models. The more correct detections and the fewer incorrect detections of the detection network, the higher the detection accuracy of the model, the lower the probability of wrong and missed detections, and the model is more suitable for practical applications.

**Figure 11:**
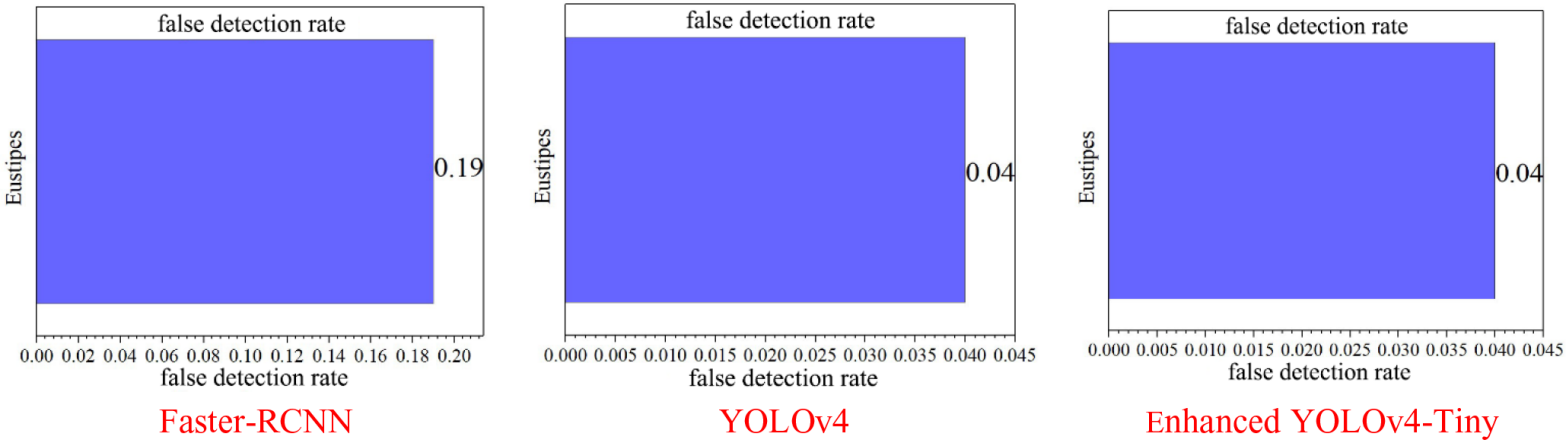
False detection rate of sugarcane stem segment recognition under different models.

**Figure 11** shows the false detection rate of the three target detection networks for sugarcane stem node recognition, and this value is an important detection index for detecting the effectiveness of network determination, and the smaller the value is, the better the detection is. In the histogram of the three target detection networks, the false detection rate of the Faster-RCNN model and the YOLOv4 model is as high as 0.19, while the enhanced YOLOv4-Tiny model has a false detection rate of only 0.04, which is one order of magnitude better than the other two network models.

The experimental results showed that the overall detection of sugarcane stem node was better for the faster detection model Faster-RCNN, but its AP value of 90.73% was lower than that of 98.68% for Yolov4 and 99.19% for the enhanced YOLOv4-Tiny. This difference was mainly in the poorer detection of broken stem node, stem node under darker light and soil-stained stem node. This may be due to the fact that Faster-RCNN does not build an image feature pyramid, and the extracted feature MAPs are single-layered and have smaller resolutions regardless of whether VGGNet or ResNet is used. Therefore, the detection accuracy is lower for broken stem node, stem node under darker lighting and stem node stained with soil. Meanwhile, Faster-RCNN uses NMS (Non-Maximum Suppression) for post-processing when RPN generates Proposal in order to avoid overlapping candidate frames with classification scores. In fact, the method is unfriendly to obscure targets, especially sugarcane in harvesting, the stem node near the roots tend to sticky soil, i.e., Proposal with two possible targets is likely to be filtered out one, resulting in missed detection.

**Figure 12:**
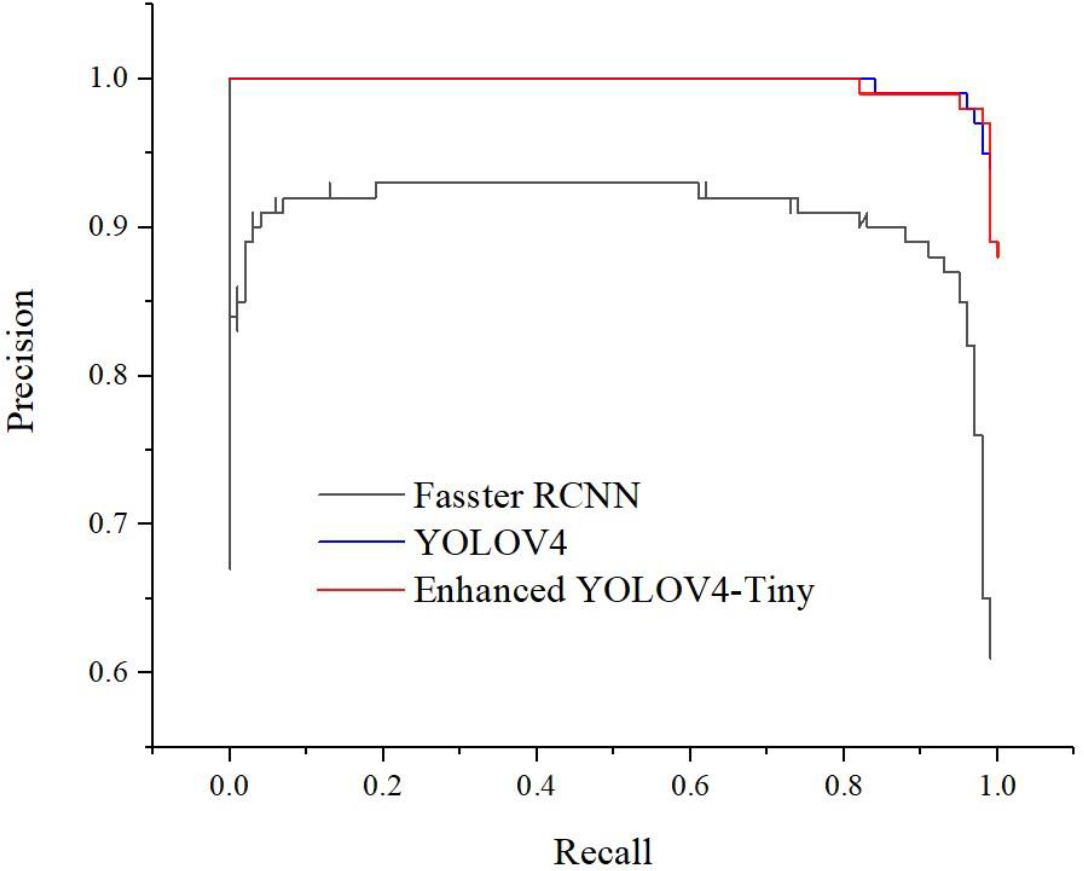
Contrast experimental PR curve.

We strictly control the relevant parameters of the experiments, use a uniform image size (608 × 608) as input, and use a uniform training and test set for testing. The final results are shown in **Table 2** and **Figure 12**. **Figure 12** shows the PR curve for each model, which is a two-dimensional curve with precision and recall as vertical and horizontal coordinates. Intuitively, it can be seen that the curve area of the enhanced YOLOv4-Tiny model and the YOLOv4 model is larger than that of the Faster- RCNN target detection model, indicating that the enhanced YOLOv4-Tiny model has higher average precision. When the Recall values of the three models were less than 0.1, the Precision values were maintained around 1.0, and the differences were not significant. However, with the increase in Recall value, the advantages of YOLOv4 and enhanced YOLOv4-Tiny models are gradually obvious, and the Precision values are very stable and do not change much.

### 3.3 Analysis of test results

**Figure 13:**
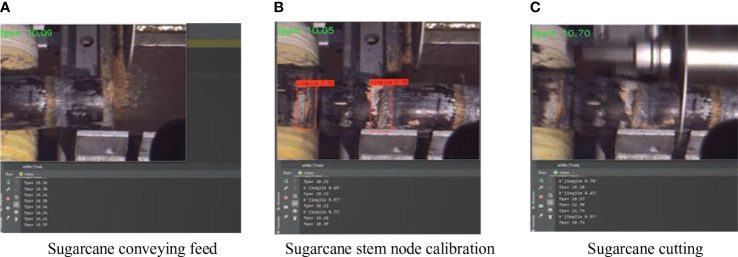
Sugarcane cutting test process. **(A)** Sugarcane conveying feed; **(B)** Sugarcane node calibration; **(C)** Sugarcane cutting.

Five black-skinned sugarcane were randomly selected from each group, for a total of 10 groups. Among the selected experimental sugarcane samples, the tops of sugarcane that could not be used as seeds, some with clods of mud or stem node already bearing damage were removed. The experiment was conducted in a place with good light conditions to reduce the effect of light on the experiment (**Figure 13**). The experiment was conducted by recording the total number of stems and the number of finished stems (divided into normal, damaged and mud-blocked stems) in each group to obtain the actual cutting rate, and the experimental results are shown in **Table 3**.

**Table 3:** Cutting accuracy.

		Actual number of node/pcs			Number of node cut/pcs
Number of groups	Normal	Damaged	With mud blocks	Normal	Damaged	With mud blocks	Rate of node recognition/%
1	78	1	1	78	1	1	98.75
2	81	0	0	81	0	0	100
3	77	0	1	77	0	0	98.71
4	84	1	0	84	0	0	98.82
5	83	1	2	83	1	1	98.83
6	76	2	2	76	0	1	96.52
7	80	1	0	80	0	0	98.76
8	79	0	0	79	0	0	100
9	82	0	2	82	0	1	98.80
10	77	1	1	77	0	0	97.46

As can be seen from **Table 3**, the accuracy of the 10 groups of experiments ranged from 96.25% to 100%, with an average accuracy of 98.64%. The enhanced model recognized 100% of normal sugarcane stem node and missed cutting for both broken sugarcane stem node and sugarcane stem node with mud lumps. The experiments showed that there were two major reasons for the occurrence of missed cuts.

(1) Sugarcane stem nodes are badly broken and the target cannot be detected.
(2) Soil obscuring sugarcane stem nodes, or misidentifying soil as stem nodes.

## 4 Conclusions and future research

In this study, an enhanced YOLOv4-Tiny model-based sugarcane stem node recognition system is designed based on the information of sugarcane stem node characteristics. The stem node target detection system realizes the acquisition and recognition of sugarcane stem node information by the device through the enhanced YOLOv4-Tiny model structure. In this paper, normal sugarcane stem nodes, broken sugarcane stem nodes and sugarcane stem nodes with soil image datasets were produced according to the actual operating environment in the field, and tested using Faster-RCNN, YOLOv4, and enhanced YOLOv4-Tiny models, respectively. The experimental results show that, compared with the Faster-RCNN algorithm and YOLOv4 algorithm, the enhanced YOLOv4-Tiny algorithm yielded an average mean accuracy (mAP) of 99.11%, a detection accuracy of 97.07%, and a transmission frame per second (fps) of 30, which can detect and identify sugarcane stem nodes quickly and accurately. After model training and experimental testing, the enhanced YOLOv4-Tiny detection model structure is better than Faster-RCNN and YOLOv4 deep learning models, and the results of this paper have important application value for advancing the development of sugarcane pre-cut seeds and promoting the development of sugarcane planting technology.

The model studied in this paper showed a missed detection of sugarcane stem nodes with soil, and future work is needed to improve the recognition accuracy of stem nodes with soil, especially those near the roots. There is a need to test the effect of lighting on the detection effect and to test the accuracy of the model for recognizing sugarcane stem nodes with different epidermal colors. In this paper, sugarcane stripped of its stems and leaves is used as the research object, which requires advance processing of sugarcane for de-stemming, and the processing is labor-intensive and inefficient. In future work, the sugarcane stem node recognition when the stems and leaves are retained can be studied.

## Data availability statement

The original contributions presented in the study are included in the article/supplementary material. Further inquiries can be directed to the corresponding author.

## Author contributions

WW: Conceptualization, Methodology, Software. KW: Supervision. PF: Grammar review. CL: Data curation, Writing- Reviewing and Editing. LT: Writing - original draft. YC: Visualization, Investigation. All authors contributed to the article and approved the submitted version.

## References

[B1] Aichen W. Xu Y. Wei X. Cui B. (2020). Semantic segmentation of crop and weed using an encoder-decoder network and image enhancement method under uncontrolled outdoor illumination. IEEE Access 8, 81724–81734. doi: 10.1109/access.2020.2991354

[B2] Aichen W. Zhang W. Wei X. (2019). A review on weed detection using ground-based machine vision and image processing techniques. Comput. Electron. Agric. 158, 226–240. doi: 10.1016/j.compag.2019.02.005

[B3] Arunabha M R. Bhaduri J. (2022). Real-time growth stage detection model for high degree of occultation using densenet-fused Yolov4. Comput. Electron. Agric. 193. doi: 10.1016/j.compag.2022.106694

[B4] Changhui Y. Zhuo W. Longye X. Yanping L. Xilong K. Wanhua Z. (.(2019). Identification and reconstruction of citrus branches in complex background based on mask r-CNN. Trans. Chin. Soc. Agric. Machinery 08), 22–30+69. doi: 10.6041/j.issn.1000-1298.2019.08.003

[B5] Changyou S. Meili W. Xinran L. Huili H. Deqiang Z. Ganran D. (2019). Identification of different types of sugarcane stem node based on machine vision. J. Comput. Appl. 04), 1208–1213. doi: 10.11772/j.issn.1001-9081.2018092016

[B6] Chen C. Zhu W. Steibel J. Siegford J. Han J. Norton T. (2020). Classification of drinking and drinker-playing in pigs by a video-based deep learning method. Biosyst. Eng. 196, 1–14. doi: 10.1016/j.biosystemseng.2020.05.010

[B7] Dihua Wu Lv S. Jiang M. Song H. (2020). Using channel pruning-based yolo V4 deep learning algorithm for the real-time and accurate detection of apple flowers in natural environments. Comput. Electron. Agric. 178. doi: 10.1016/j.compag.2020.105742

[B8] Fangfang G. Fu L. Zhang X. Majeed Y. Li R. Karkee M.. (2020). Multi-class fruit-on-Plant detection for apple in snap system using faster r-CNN. Comput. Electron. Agric. 176. doi: 10.1016/j.compag.2020.105634

[B9] Fu H. Song G. Wang Y. (2021). Improved YOLOv4 Marine Target Detection Combined with CBAM. Symmetry 13, 623. doi: 10.3390/sym13040623

[B10] Huaning S. Peng J. Tuo N. Xia H. Peng Y. (2021). Study of sugarcane buds classification based on convolutional neural networks. Intelligent Automation Soft Computing 27, no. 2, 581–592. doi: 10.32604/iasc.2021.014152

[B11] Masum B. Wang X. Yu J. Jiang Yu (2022). Real-time goat face recognition using convolutional neural network. Comput. Electron. Agric. 194. doi: 10.1016/j.compag.2022.106730

[B12] Rui Y. Li J. Liu Qi Huang W. Yin K. Qiao Xi. (2020). Gradient-based method for the identification of multi-node in sugarcane. Inf. Process. Agric. 7, 491–499. doi: 10.1016/j.inpa.2020.01.004

[B13] Rui S. Li T. Yamaguchi Y. (2020). An attribution-based pruning method for real-time mango detection with yolo network. Comput. Electron. Agric. 169. doi: 10.1016/j.compag.2020.105214

[B14] Shangping Li Xianghui Li Ke Z. Kaihua Li Honglei Y. Zongxiao H. (2019). Improved YOLOv3 network to improve real-time dynamic identification efficiency of sugarcane stem node. Trans. Chin. Soc. Agric. Eng. 23, 185–191. doi: 10.11975/j.issn.1002-6819.2019.23.023

[B15] Shenglian Lu Chen W. Zhang X. Karkee M. (2022). Canopy-Attention-Yolov4-Based Immature/Mature apple fruit detection on dense-foliage tree architectures for early crop load estimation. Comput. Electron. Agric. 193. doi: 10.1016/j.compag.2022.106696

[B16] Shiping Z. Jiaxin Z. Guanglin H. H. &Li (2020). Wheat grain integrity image detection system based on CNN. Trans. Chin. Soc. Agric. Machinery 05), 36–42. doi: 10.6041/j.issn.1000-1298.2020.05.004

[B17] Subramanian P. Selvi S. T. (2021). Detection of maturity stages of coconuts in complex background using faster r-CNN model. Biosyst. Eng. 202, 119–132. doi: 10.1016/j.biosystemseng.2020.12.002

[B18] Wang D. Su R. Xiong Y. Wang Y. Wang W. (2022). Sugarcane-Seed-Cutting System Based on Machine Vision in Pre-Seed Mode. Sensors 22, 8430. doi: 10.3390/s22218430 36366128 PMC9655777

[B19] Weikuan J. Tian Y. Luo R. Zhang Z. Lian J. Zheng Y. (2020). Detection and segmentation of overlapped fruits based on optimized mask r-CNN application in apple harvesting robot. Comput. Electron. Agric. 172. doi: 10.1016/j.compag.2020.105380

[B20] Weizheng Z. Weiwei Z. Huanlong Z. Qiqiang C. ChenChen D. (2017). Identification and localization of sugarcane stem node based on hyperspectral imaging technology. Chin. J. Light Industry 05), 95–102. doi: 10.3969/j.issn.2096-1553.2017.5.013

[B21] Wen C. Ju C. Li Y. Hu S. Qiao Xi (2021). Sugarcane stem node recognition in field by deep learning combining data expansion. Appl. Sci. 11, no. 18. doi: 10.3390/app11188663

[B22] Xiaoyu Li Du Y. Yao L. Wu J. Liu L. (2021). Design and experiment of a broken corn kernel detection device based on the YOLOV4-tiny algorithm. Agriculture 11, no. 12. doi: 10.3390/agriculture11121238

[B23] Xie D. Chen L. Liu L. Chen L. Wang H. (2022). Actuators and Sensors for Application in Agricultural Robots: A Review. Machines 10, 913. doi: 10.3390/machines10100913

[B24] Xuelong Hu Liu Y. Zhao Z. Liu J. Yang X. Sun C.. (2021). Real-time detection of uneaten feed pellets in underwater images for aquaculture using an improved YOLO-V4 network. Comput. Electron. Agric. 185. doi: 10.1016/j.compag.2021.106135

[B25] Xu Li Pan J. Xie F. Zeng J. Li Q. Huang X.. (2021). Fast and accurate green pepper detection in complex backgrounds via an improved Yolov4-tiny model. Comput. Electron. Agric. 191. doi: 10.1016/j.compag.2021.106503

[B26] Yiqi H. Jisen H. Rui Y. Wenshan H. XiaoBo W. (2017). Height detection and test of sugarcane cutting based on machine vision. J. Chin. Agric. mechanization 09), 81–87. doi: 10.13733/j.jcam.issn.2095-5553.2017.09.017

[B27] Zhenfeng D. Yongxiu X. Yangrui L.. (2022). Future prospect of microbial fertilizer application in sugarcane. Guangxi Sugar Industry 42 (02), 20. doi: 10.3969/j.issn.1007-4732.2022.02.004

